
JOURNAL INFORMATION
==============================
NLM Title Abbreviation: Med Chir Rev
NLM Journal ID: 0034617

The Medico-Chirurgical Review

Publisher: Biomedical Journal Digitization Project

ARTICLE INFORMATION
==============================
PMCID: PMC4966190
PMID: 29917612
Article version: 1
Subjects: Reviews

Prize-Memoir on White-Swelling

Print publication date: 1828 Jul 1
Volume: 9
Issue: 17
First page: 42
Last page: 48
1828 Apr 26
License: This work is free of known copyright restrictions. For more information, please see PMC Back Issue Digitization.
License URL: https://creativecommons.org/publicdomain/mark/1.0/

==============================

### V.

### *Prize Memoir on "White Swellings" of the Joints.* By Dr. Nicolai, of Berlin.*

We had imagined that the term "White Swelling" was pretty well banished from the surgical nosology, since the appearance of Mr. Brodie's work on the Diseases of the Joints. Mr. Brodie proved most satisfactorily that, under the above designation, were confounded diseases distinct and distinguishable from each other, having different causes, courses, and terminations, and requiring very different remedies for their cure or their treatment. The four principal diseases to which the textures, soft and hard, of the joints are subject, were described by Mr. B. under the heads of Inflammation of the Synovial Membrane, the most common of all—Morbid Change of Structure of the Synovial Membrane, comparatively rare—Ulceration of the Cartilages—and, lastly, Scrofulous Caries of the Articulating Extremities of the Bones. Now, to jumble up these various affections under the vague term of white-swelling, must be, in the first place, unphilosophic, and, in the second, unsafe; because it leads us to employ a farrago of remedies indiscriminately in all cases, in some of which they are useless, and in others may be worse. This censure, however, is not fairly applicable to the Memoir of Dr. Nicolai; for, although he makes use of the objectionable term, he confines it to a particular affection, originating *external* to the joints, and which has never been described, as far as we know, by other authors.

The Doctor divides the "true white-swelling" into the acute and chronic forms, of which the latter appears to be the most common. It presents, according to our author, three tolerably well-marked stages, each characterized by different symptoms, and having different anatomical characters on dissection.

1*st Stage.* The disease commences by a sensation of lassitude in the limb, and pain in the joint, increased on motion. The pains shift their seat, and are compared to what young persons commonly call "growing pains." The pain is not increased, but rather relieved, by pressure and ligatures around the joint. After a time, the lassitude and pain on motion become augmented, particularly in bed, preventing sleep, and giving the patient much uneasiness. On examination, there is seen no tumefaction, no discolouration, but the temperature of the surface is somewhat raised, and there is a kind of crepitus distinguishable in the joint. The patient, on inquiry, will very frequently be found to have suffered from *rheumatic pains.*

2*d Stage.* After the above symptoms have persisted for some weeks, the joint begins to swell, particularly at the point where the greatest pain was experienced. The swelling is soft, elastic, and gradually extends to the whole joint, whilst the skin becomes stretched and shining, and motion is painful and difficult. The pain is now severe, and, when the knee is the part affected, it is felt particularly in the ham, obliging the patient to keep the leg constantly bent. The enlargement goes on increasing, the veins become distended, and the limb emaciates greatly.

3*d Stage.* The tumefaction having reached its highest degree, the skin reddens at different points, where it becomes prominent and thin, whilst, at the same time, an indistinct fluctuation is apparent. The limb grows œdematous, and, where the pain is most severe, if an opening is made with a lancet, or ulceration takes place, a bloody puriform fluid is evacuated. If a probe be introduced, the bone, tendon, or ligaments, are found exposed, and the instrument passes with facility in any direction, shewing that the parts which surround or compose

* The Essay was written in Latin, and crowned by the "Cercle Medical" of Paris.

the joint are destroyed, or perforated with sinuses. Hectic is now fairly established, and if the disease be allowed to remain, the patient gradually sinks.

So much for the symptoms of "white-swelling," let us now look at the pathology. In the first stage of the disease, we are informed by Dr. Nicolai that the cellular tissue surrounding the tendons and ligaments is loaded with blood-vessels, thickened, and infiltrated with a great quantity of a mucous or "glutinous" matter, which matter is principally deposited immediately around the tendons and ligaments. In the knee, this coagulable lymph, or whatever else it may be, is principally seated at the posterior part of the articulation, at least in the first instance; in the hip, it exists at the lateral and posterior parts of the joint, and, in the foot, at the side of the malleoli. The deep parts of the articulation have, as yet, experienced little change, save that the periosteum and synovial membrane are injected in parts, whilst the latter is altered in consistence, and adherent to the mass of lymph investing the ligaments on its outside.

In the *second stage*, the vascular condition of the cellular tissue has disappeared, and the latter structure has become converted into a fibrous, lardaceous substance, intersected by white striæ, which, on examination, are found to be made up of enlarged vessels, mostly obliterated, and so thin in their coats as to resemble veins. This fibrous substance envelopes the ligaments, &c. but it can be readily peeled from them, leaving them in their natural condition, and "proving that the original site of white-swelling is in the cellular texture." Some few points again are much more vascular, of a blackish red colour, and infiltrated with caseous and livid looking matter.

*3d Stage.* The principal vessels in the tumour are now obliterated—the skin, cellular substance, &c. are more or less disorganized—the bones, tendons, and ligaments, are enveloped in a caseous, lardaceous matter—the synovial capsule is red and thickened, and its inner surface lined with a caseous matter. When sinuses exist, they ramify through the soft parts in various directions, penetrating to, and denuding of periosteum the bones, or exposing the cartilages.

Such is the description given us of the *chronic* white-swelling; the *acute* is usually preceded by rheumatic fever—the joint is *red*, tumefied, and very painful on the slightest touch—the pain is aggravated when the patient is warm in bed—the skin is glistening and burning hot, and there may be a collection of fluid within the joint. After the lapse of a few days, the febrile symptoms diminish or disappear, the acute symptoms subside, and the disease ultimately takes on the chronic character above-described.

Our author concludes that the seat of the disease is in the cellular membrane which invests the ligaments and tendons, and connects the skin with the deeper parts—that the matter deposited in these situations is the result of chronic inflammation, being at first common lymph, afterwards more resembling gelatine, and finally, becoming organized, and constituting fibrous and lardaceous matter. This affection is more common in youth than in mature age, "in men than in women," the latter of which positions we are very much inclined to doubt. Every thing which weakens the body, whether bad health or bad diet, disposes to the disease, which is very prevalent amongst the poor, especially of Great Britain, "where they live upon oaten bread and a great many potatoes."* If

* This must be alarming intelligence to the Irish, ruddy-faced and stout-limbed as they look. The fact is, however, that it would be well for the poor of Great Britain, and especially the miserable wretches that people some of our agricultural districts, if they *could* get a sufficiency of the much calumniated "mealy potatoes." It is not the *kind* of food, unfortunately, but the *want* of it, that is the cause of so much disease amongst our poor.

the disease has reached its height, it is very rarely cured, but if brought under care at the commencement, the treatment is generally successful. White-swelling is seldom or never cured without the occurrence of anchylosis, and it is to the attainment of this that we must mainly direct our efforts. Our author goes on to describe inflammation of the synovial membrane and other affections, which may be mistaken for white-swelling; he, also, gives an elaborate description of what he terms *arthrocace*, but which is nothing more than the "scrofulous caries of the bones" so ably treated of in Mr. Brodie's work. These subjects we shall pass over at once, as no novelty can possibly attach to them; but we shall take the liberty of offering a few remarks on that part of Dr. Nicolai's Memoir which appertains to what he has designated true white-swelling, and which we have so fully described.

It will be observed that the Doctor makes the disease originate not *within* the joint, but *without* it, to wit, in the cellular membrane beneath the skin, and investing the ligaments or tendons. Most certainly this is not the generally received opinion, and we are very much inclined to doubt if it be the correct one. We have seen very many cases of white-swelling, but we must confess that we never saw any where the appearances on dissection have led us to imagine that the mischief had *commenced* on the outside of the capsule. Most undoubtedly as the disorganization advances in the joint, whether it be erosion of the cartilage, or caries of the bone, changes do take place in the cellular tissue, but then they are only secondary, and not of that importance which Dr. Nicolai has assigned to them. In short, we believe that, in the very great majority of those chronic diseases of the joints, which almost exclusively people our hospitals, the morbid action originates, *bonâ* fide, *in* the joint, and nowhere else. In the details of symptoms with which our author has supplied us, he remarks that the patients, for the most part, have laboured under rheumatism, and this is a point to which we would especially wish to draw the attention of our readers, as we think it of considerable consequence. A sailor, whilst in the East Indies, became subject to acute rheumatism, and was obliged to return to Europe. He entered St. George's Hospital with an enlargement of the left knee, which was in a state of semi-flexion and incapable, or nearly so, of motion. There was apparently little effusion in the joint, but there was pain on pressure or motion, with a very curious crepitation, exactly resembling that produced by erosion of the cartilage, and the contact of portions of exposed bone. A great variety of remedies, local and constitutional, were employed, and, after eight or ten months, he left the Hospital, very little better than when he entered it. This is not a solitary case, in fact every practitioner must have seen very many where the symptoms are barely to be distinguished from those of the more formidable affections of the joints, but where the patient almost invariably gets off with a stiff limb. Are we to suppose that the periosteum is actually altered, the cartilages destroyed, or may we not rather conclude, that the cellular tissue investing the ligaments, tendons, and synovial bag, is the seat of the rheumatic inflammation? However this may be with the *chronic* rheumatic affections of the joints, we are satisfied that in some *acute* cases, the disease does most unequivocally originate in the parts without, and spread, rapidly and fatally, to those within.

*Case* 1.* A prostitute, who had been subject to rheumatism, was admitted into St.

---

* This and the following case have been recorded in the Medical Gazette, but we had an opportunity of seeing them ourselves, and as they illustrate our remarks, we shall make no apology for briefly introducing them. The succeeding cases have not been published elsewhere.

George's Hospital, under the care of Mr. Brodie, with most extensive swelling, reaching from the upper part of the right thigh over the knee, for some little distance down the leg. The swelling was diffuse, elastic, exquisitely painful upon pressure, and of a glossy marbled white, in fact, having all the characters of rheumatic inflammation of the cellular tissue, and probably of the periosteum. At this time the knee appeared unaffected in itself, although the soft parts around it were involved in the enlargement. She had been subject to rheumatism, was leading a life of prostitution, and all this enormous swelling, and excessive pain, had appeared suddenly the day before her admission. The limb was leeched and blistered, and the mouth made sore with calomel and opium, with the effect of almost entirely dispersing the pain and swelling. However, when all promised well, symptoms of ulceration of the cartilages of the knee-joint set in, and of this disease the poor creature shortly died. On dissection, the cartilages investing the condyles of the femur, the head of the tibia, and the patella, were ulcerated, and the cancelli of the bones exposed beneath. The periosteum was easily separable from the thigh-bone for some distance up, and the bone itself was vascular.

*Case* 2. This was as complete a tally as possible with the one we have just recorded. A washerwoman was admitted into St. George's Hospital, under the care of Mr. Keate, with precisely the same kind of diffuse swelling, extending from the upper part of the thigh, over the knee, and down the leg. There was the same excruciating pain on pressure or motion—the same elasticity—the same opake and remarkably bloodless hue, making the limb look as if it belonged to a statue rather than a living woman. These symptoms had come on suddenly twelve days previously, and the disease ran a similar course with the preceding, save that matter formed in the cellular tissue of the ham, and was evacuated. At last the patient died, worn out by pain and hectic, and on examining her body, the cartilages of the knee-joint were found extensively ulcerated. The inflammation, however, in the cellular texture had gone on to suppuration, for the thigh-bone and its periosteum were bathed in one large sloughy abscess from condyle to trochanter.

Here, then, are two cases of rheumatic inflammation, evidently originating in the subcutaneous and deeper cellular texture, and spreading thence to the cartilages of the knee-joint, the periosteum, and the bone. If this order of things can take place, when the rheumatic inflammation is of the acute kind, why, we ask, should it not, when the action is more chronic? Rheumatism appears to have an almost exclusive *penchant* for the cellular tissue and the textures that are formed from it, viz: the periosteum—the ligaments—the tendons—the articulating capsules, and the investing membranes, as the pericardium. Another well-known law in the etiology of rheumatism, is its disposition to attack those parts which are especially exposed to atmospheric influence, and to leave those untouched which are well clothed and warmed by fat and muscle. Hence, its greater prevalence in the knee and ankle, its comparative rarity in the hip. We are willing to allow that it not unfrequently does assail the deeper structures, the pericardium, for instance, but then it is by that peculiar action of which we talk so much and know so little—metastasis. Granting then, that rheumatic inflammation has its seat in the cellular tissue, and more especially in the portions of that tissue which are most superficial and exposed, we think it extremely probable, that in the chronic enlargements of the joints *after rheumatism*, constituting the *rheumatic* white-swelling,* Dr. Nicolai's description is correct, and the disease very frequently has its *origin* external to the joint. In practice, perhaps, this may not signify very much, but then correct pathology is at all times desirable; first, because it gives more certainty to our diag-

* We confine it to the rheumatic form, and are quite ready to allow, that even in this the synovial membrane is not unfrequently primarily affected; indeed, we think it probable, that this membrane, when not the first, is amongst the first to be attacked in the "rheumatic white-swelling."

nosis and prognosis, and secondly, because without it we are mere empyrics, groping in the dark, and employing remedies for no reason but that others, or ourselves, have employed them before.

We now advert to the treatment recommended by Dr. Nicolai. In the inflammatory stage he applies leeches, lotions, &c. to the part, and gives saline medicines internally; when inflammation has passed away, frictions with the camphorated mercurial ointment and volatile liniments, combined with light doses of the submuriate of mercury and jalap. An aperient of this kind should be given every second day, whilst the disease is kept at bay by local abstractions of blood, blisters, or the application of the actual cautery. In the second and third stages, when there are no open ulcers, we are recommended to employ gentle pressure by bandages, straps, the empl. hyd. c. ammoniac, etc., combining with these, according to circumstances, baths, frictions, the internal use of tartarised antimony, digitalis, sarsaparilla, and a variety of other general means. As a dernier resort we must amputate, but even in this case, we are told that death is frequently the consequence.

From this it will be seen, that our author's methodus medendi is meagre enough. In the acute cases of rheumatic inflammation of the thigh, terminating in ulceration of the cartilages of the knee, of which we have detailed two cases, Mr. Brodie is in the habit of putting the patients, as speedily as possible, under the influence of calomel and opium. In one case, that of a page in a nobleman's family, this plan was completely successful, and, upon the whole, Mr. B. has seen more good result from it than any other. In the chronic forms, which are by far the most common, we fancy, local measures will be found most advantageous. The alternation of blisters, and the antimonial plaster, we have seen of considerable service, but caustic issues are not attended with the same advantage in these cases, as in the true idiopathic ulceration of the cartilages. The application of the moxa is of use, particularly in the after stages, where there is rigidity of the tendons, &c., and we believe it will be found that caustic issues are of service, when the disease is in the deep-seated structures—the moxa, when it has its seat in the more superficial textures. Perfect rest, of course, is indispensible, and this is best obtained by means of pasteboard splints, moulded to the form of the joint, and covered with wash-leather, forming a very effectual and indeed a very *neat* apparatus. There is a mode of applying pressure and preventing motion, which we have seen practised by Mr. Brodie, and which answers, in many instances, exceedingly well. It is this. The joint is enveloped with strips of adhesive plaster, and then a few turns of a stout calico roller are applied; over these, fresh strips of plaster, then more turns of the roller, and so on, forming a perfect *case* of bandage and strapping, which effectually checks all motion, and, by keeping the joint warm and moist with its perspiration, answers the purpose of a vapour-bath.

There is another affection of the joints, for the knowledge of which we are indebted to Mr. Brodie, although he has not as yet given any detailed account of it to the public.*

*Case.* Mary Moore, æt. 20, was admitted into St. George's Hospital, on the 4th April, 1827, under the care of Mr. Brodie. She stated, that about two months prior to her admission, she met with a fall upon the left elbow-joint, producing considerable blackness and some swelling, which, however, under the use of soap-liniment and bandages, gradually subsided. There was much pain, which continued but little relieved, with some swelling of the joint, and a host of anomalous symptoms.

---

* At page 338 of the 2d edition of Mr. Brodie's work upon the Joints, the subject is alluded to.

On her admission, she presented the following symptoms; the joint, if anything, swollen—the fore-arm semi-flexed—the fingers and thumb firmly contracted. There was considerable "shooting" pain in the elbow-joint, stretching down to the wrist and hand, but the principal seat of pain was on the fore-part of the joint, and appeared to be seated in the tendon of the contracted biceps muscle. The pain was brought on by attempting to bend or extend the joint, by supination and pronation, in short, by motion in any way; it was also brought on by pinching, or even lightly *tapping* the *skin* in the neighbourhood, and even, as she stated, by rising suddenly from her seat, or any unexpected noise, as the opening of a door. On endeavouring to straighten the finger, a regular paroxysm of hysteria was the consequence. She complained also of her hip, which was not swollen, but painful, the pain shooting down to the inside of the foot and toes. Pressing on the heel, and by that means pushing the head of the bone into the socket, gave much pain—so did any attempt at inverting the foot, whilst she could bear eversion a great deal better. Pressure upon the trochanter, or pinching the skin brought on the pain, and if the pressure was at all considerable, a kind of hysterical convulsion succeeded. If, however, at the time of making the examination, *the girl's attention was drawn off, she seemed not to suffer at all.* Her rest at nights was disturbed, but she had no hectic symptoms, nor was her appearance at all indicative of severe disease. The catamenia were irregular, and she was subject to hysterical affections.

These were the symptoms presented by the patient, and what did they indicate? ulceration of the cartilages? Certainly there were many of the characters of that disease; as, the appearance of the symptoms after the reception of a blow—the little swelling—the severe pain, shooting down the limb, and increased on motion of the joint, or pressure. There were, however, other symptoms, which gave strong reason to believe that the disease was by no means of so serious a nature. The absence of hectic—the appearance of health—the pain aggravated on merely pinching the integument, or any sudden noise—the circumstance of both the hip and elbow being affected, and the decided nervous paroxysms, led Mr. Brodie to believe that this was a case of hysteria, simulating ulceration of the cartilages. The girl was accordingly ordered tincture of valerian and vinum alois, with active purges, and the daily use of the shower-bath. In little better than a month, she was so far improved as to be made an out-patient, and a short time ago we learnt, that she had had a child, and was perfectly recovered!

*Case* 2. This was an instance of the same affection occurring in the knee-joint. Mary Kenrick, æt. 18, was admitted into St. George's Hospital, Nov. 14th, 1827, under the care of Mr. Brodie, labouring under the following symptoms. The joint was little swollen, exceedingly painful, and incapable of motion, at least by her own efforts, without considerable suffering. The pain did not awake her from her sleep at night, and there was a good deal on pinching the integument. The bowels were costive—the catamenia irregular—the appearance strong and lusty. Two years previously she received a blow upon the knee, which swelled, and was very painful. She was treated by caustic issues, and afterwards getting plenty of air and exercise, quite recovered. Six months prior to admission, she again received a blow, and from that instant her present symptoms had continued. She was ordered pil. alois c. myrrhâ, and briskly purged; subsequently she took valerian and vinum alois, and, on the 18th December, was made an out-patient, having little the matter with her.

We could detail several other cases of the same kind, but it would be unnecessary, as the above are fair samples of the affection. It occurs in unmarried hysterical females, and is no doubt very frequently mistaken, and treated for organic disease. We think, from what we have observed, that it is most common in the knee, that joint being most exposed to those little accidents and injuries, which may be said to be the exciting causes of the complaint. The symptoms very strongly resemble those of ulceration of the cartilages, but, on accurate investigation, there will be found the following points of difference between them. *In ulceration of the cartilages,* the pain is worse at nights, is accompanied by startings of the limb, and wakes the patient from her sleep. *In the hysterical affection*, the pain may prevent the patient's falling to sleep, but very seldom indeed awakes her, *when* asleep. *In ulceration of the cartilages*, the pain is aggravated on motion of the joint; *in this*, not only is it increased by motion, but even by *tapping* the integument, or any impression on the general nervous system, as a sudden noise. *Ulceration of the cartilages* is a serious disease, and the system knows that it is so, for there is ill-health, hectic, emaciation; but the hysterical affection is *not* a serious disease, and the patient is, for the most part, rather sleek and full-blooded than worn out by fever or irritation. The catamenia are generally irregular—the bowels costive, and other hysterical symptoms are mostly present, to mark the *bent* of the patient's constitution. There is one circumstance attending these hysterical affections of the joints which is calculated to mislead practitioners, namely, their being, for the most part, consecutive to a blow or injury. The fact is, that it is generally some cause of this kind which determines the *local* habitation of the hysterical leaven that is lurking in the constitution. Thus, if a nervous girl be bled, she will have spasmodic twitchings of the muscles of the arm, and every symptom of wounded nerve—if she be a young lady, wearying the muscles of her back at the harp or piano-forte, or "doomed for a certain time" every four and twenty hours, to occupy one of those modern instruments of torture, "education chairs," the spine will probably be the part affected; and again, if the patient be in the lower walks of life, exposed to injury of various parts, those, whatever they may be, will become the seat and centre of the nervous malady. We have heard Mr. Brodie declare, that he has frequently been himself mistaken in his diagnosis of these hysterical affections of the joints, and he has seen instances, where unfortunate young ladies have been confined for years on mattrasses and planes for disease of the spine, when the complaint was solely nervous, and there existed not a particle of disease in the vertebral column!

With regard to the treatment, Mr. Brodie is in the habit of making light of the disease, and diverting the attention of the patient from it. A girl, for instance, had obstinate abscesses in the groin, and was subject to hysteric fits. She was discharged the hospital, but the sores becoming worse, was re-admitted, with this proviso, that she was to have no hysterics! She never had a paroxysm. Another girl was admitted with the hysterical affection of the knee, and a few days afterwards, on asking the neighbouring patients whether she was ever awaken in the night by the pain, they declared not. Mr. Brodie commented on this as a favourable symptom in the girl's hearing, and, on his next visit, was surprised to find that the patients near her could scarcely get a wink of sleep on account of her screams during the night! Mr. B. very quietly told the girl that he should not allow of any thing of the kind, and from that instant her nocturnal sufferings were lulled! When the menstruation is irregular, the vinum alois is useful—active purging is almost always of great service. If the patient has a languid circulation, with cold hands and feet, &c. Mr. Brodie employs the following form:—

Tinct. ferri ammon. ʒj. Ammon. subcarb. grs. v. Infus. quassiæ, Aquæ cinnamom. āā ʒvi. ℳ. ft. haustus bis die sumendus.

The tincture of valerian is also of considerable service; and, as a local application, when the pain is at all severe, a rag, dipped in a compound of an ounce and a half of spirit of rosemary, to six ounces and a half of camphor mixture.
